# Preparation and Phase Change Performance of Graphene Oxide and Silica Composite Na_2_SO_4_·10H_2_O Phase Change Materials (PCMs) as Thermal Energy Storage Materials

**DOI:** 10.3390/ma13225186

**Published:** 2020-11-17

**Authors:** Wen Tao, Xiangfa Kong, Anyang Bao, Chuangang Fan, Yi Zhang

**Affiliations:** 1 School of Material Science and Engineering, Anhui University of Technology, Anhui 243002, China; wentaowz@sina.com (W.T.); kongxiangfa6@sina.com (X.K.); baoay1999@163.com (A.B.); chgfan@ahut.edu.cn (C.F.); 2 Key Laboratory of Green Fabrication and Surface Technology of Advanced Metal Materials (Anhui University of Technology), Ministry of Education, Anhui 243002, China

**Keywords:** graphene oxide, phase change materials, inorganic hydrate salt, supercooling, thermal energy storage

## Abstract

In this study, a novel nucleating agent composed of graphene oxide (GO) and silicon dioxide (SiO_2_) (GO–SiO_2_) is developed. GO is used as a skeleton material through which SiO_2_ nanomaterials are absorbed and subsequently incorporated into Na_2_SO_4_·10H_2_O phase change materials (PCMs). Furthermore, this study examines the phase change performance of the composite Na_2_SO_4_·10H_2_O materials. Fourier-transform infrared (FTIR) spectra confirmed the physical combination of GO with a SiO_2_ nanoparticles. Scanning electron microscope (SEM) results showed that the GO–SiO_2_ composite exhibited a layered structure and excellent dispersibility. The GO–SiO_2_ composite Na_2_SO_4_·10H_2_O PCMs displayed a low level of supercooling, i.e., about 1.2 °C with the addition of GO–SiO_2_ at 2.45 wt%. This was because the synergistic relation of the GO and the high dispersion SiO_2_, imparted more nucleation sites for Na_2_SO_4_·10H_2_O. Additionally, the prepared PCMs achieved high phase change latent heat and thermal conductivity, even under these conditions. The results show that the GO–SiO_2_ in the Na_2_SO_4_·10H_2_O exhibited advantageous application prospects for the improvement of the thermal performance of hydrate salts.

## 1. Introduction

Phase change materials (PCMs) are a kind of thermal energy storage material that can provide a high density of heat storage within a small temperature range, i.e., nearly that of the phase change temperature. This characteristic of PCMs makes them suitable for a wide range of applications in solar energy production, building energy conservation, refrigeration logistics, power systems, waste heat recovery, heating and air conditioning, household appliances and so on [1,2]. Inorganic hydrate salts are a kind of PCM that feature characteristics including high phase change latent heat, nonflammability, low cost and high thermal conductivity [3,4]. Compared with organic PCMs, inorganic hydrated salts have demonstrated many potential applications in the field of thermal energy storage [5,6]. However, there are also problems with inorganic hydrate salts, notably their phase separation and level of supercooling, which have become the key issues restricting the application of these kinds of materials [7,8].

The addition of a nucleating agent is an effective way to overcome the low degree of supercooling of inorganic hydrate salts [9,10]. The adsorption of inorganic hydrate salts into porous materials can also reduce the degree of supercooling [11,12,13]. Na_4_P_2_O_7_·10H_2_O can be used as a nucleating agent to reduce the required degree of supercooling of some hydrate salts [14], while other materials, like SrCl_2_·6H_2_O, can be used as nucleating agents of CaCl_2_·6H_2_O due to the common crystal structure of the two salts [15]. Liu et al. [16] experimented with Na_2_B_4_O_7_·10H_2_O, using nano-Al_2_O_3_ as a nucleating core; the results showed that nano-Al_2_O_3_ acted as nucleating core, and that the supercooling degree could be effectively reduced from 7.8 to 1.6 °C. Expanded graphite (EG) is a kind of carrier material with a porous structure; the adsorption of hydrate salts into the inner space of EG could prevent the leakage of PCMs in the phase change process. Xiao et al. [17] inserted Ba(OH)_2_·8H_2_O into EG; their results showed that the supercooling degree of Ba(OH)_2_·8H_2_O can be reduced from 13 to 2.4 °C. Liu et al. [18] used bentonite as a supporting material of CH_3_COONa·3H_2_O, finding it to be helpful in solving the leakage problems of CH_3_COONa·3H_2_O PCMs.

The addition of nanoparticles is not only effective in improving the thermal performance of PCMs; supercooling problems can also be solved in this fashion [19,20,21]. As a kind of supporting material, nano-SiO_2_ presents a porous structure with massive mesoporosity in its inner space, such that PCMs can be well adsorbed therein [22]. Peng et al. [23] used fumed SiO_2_ as a nucleating agent; their results showed that the supercooling degree of disodium hydrogen phosphate dodecahydrate (Na_2_HPO_4_·12H_2_O) could be reduced from 14.4 to 4.1 °C when 2 wt% of fumed SiO_2_ was added. As a kind of two-dimensional nanomaterial, graphene oxide (GO) has been proven to be effective in improving the thermal properties of PCMs. Xu et al. [24] used SrCl_2_·6H_2_O and GO as nucleating agents to reduce the supercooling degree of CaCl_2_·6H_2_O, and found that a percentage reduction in supercooling of about 99.2% could be achieved when 0.8 wt% SrCl_2_·6H_2_O and 0.02 wt% GO were admixed. Xia et al. [25] prepared poly(ethylene glycol) (PEG) using GO nanosheets; their results showed that PEG homogeneously intercalated into GO to form composite PCMs with a lamellar structure, resulting in highly thermally conductive and reliable PCMs.

As a kind of commonly used inorganic hydrate salt, sodium sulfate decahydrate (Na_2_SO_4_·10H_2_O) possess a phase change temperature of about 32.4 °C and a phase change latent heat of about 254 J/g. This approximate phase change temperature makes it a promising material for energy storage in buildings. However, the supercooling and phase separation of Na_2_SO_4_·10H_2_O also present problems that limit its practical applications [26]. The high dispersibility of SiO_2_ nanoparticles is beneficial for the reduction of the degree of supercooling when combined with Na_2_SO_4_·10H_2_O PCMs. GO also exhibits high compatibility with Na_2_SO_4_·10H_2_O PCMs and good thermal conductivity; this is the trend observed in the development of the nanomaterials that are used in inorganic hydrate salts. Nano-nucleating agents must have a high specific surface area to ensure the dispersal of nanomaterials in hydrate salts, and the hydrate salts should have a better phase change energy storage performance when the nanomaterials are incorporated. Currently, there is no research focusing on the nucleating agent composed of SiO_2_ and GO and its composite in the hydrate salts. In this study, in order to investigate the nucleating effect of nanoparticles in Na_2_SO_4_·10H_2_O, SiO_2_ nanoparticles were synthesized through the sol–gel method. Through the composition of GO with the prepared nano-SiO_2_, GO–SiO_2_ composites were developed. Their chemical structure, micromorphology and specific surface area were experimentally studied. Also studied was the influence of GO–SiO_2_ on the supercooling degree, crystallization behavior, phase change performance and thermal conductivity of Na_2_SO_4_·10H_2_O.

## 2. Materials and Methods

Sodium sulfate decahydrate (Na_2_SO_4_·10H_2_O, SSD, analytical grade) was used as the PCM, supplied by Aladdin Chemical Reagent Co., Ltd. (Shanghai, China). Tetraethoxysilane (TEOS, analytical grade) provided by Sinopharm Chemical Reagent Co., Ltd (Shanghai, China), was used as silica precursor. Formamide (analytical grade), supplied by Sinopharm Chemical Reagent Co., Ltd. (Shanghai, China), was used as organic solvent. Graphene oxide (GO) aqueous dispersion with a solids content of 7.5 g/L was supplied by Jiangnan Graphene Research Institute (Changzhou, China).

SiO_2_ nanoparticles were synthesized under standard conditions using formamide solution as a solvent and TEOS as a silica precursor [27]. A certain amount of TEOS was mixed in formamide and stirred in a water bath at about 35 °C. NH_3_·H_2_O (28 wt%) was added and the pH value of the solution was adjusted to 11. The mixture was stirred for 24 h, filtered and dried to obtain the SiO_2_ nanoparticles. The prepared SiO_2_ and GO were mixed and ultrasonically dispersed for 2 h; then, the mixture was filtered and dried to obtain the GO–SiO_2_ composites. Na_2_SO_4_·10H_2_O and GO–SiO_2_ were mixed and magnetically stirred at 45 °C for 0.5 h to obtain the composite PCMs. The mix proportions of the GO–SiO_2_ composites and the composite PCMs are shown in Table 1.

The chemical structures of the samples were obtained by Fourier-transform infrared spectroscopy (FTIR, Nicolet 6700, Thermo Scientific, Waltham, MA, USA) with KBr pellets with a wavenumber of 4000 to 400 cm^−1^ and a scanning speed of 10 cm^−1^/s. The nitrogen adsorption–desorption isotherms of the prepared SiO_2_ and GO–SiO_2_ composites were determined by a pore size analyzer (Micromeritics, Asap2460, Norcross, GA, USA). The crystalline behavior of the composite PCMs was characterized by means of X-ray diffraction using an automated X-ray powder diffractometer (XRD, D8 Advance, Bruker, Leipzig, Sachsen, Germany) with CuKa radiation (λ = 1.5418 Å). The surface morphologies of the composite PCMs were observed using a field emission scanning electron microscope (FESEM; FEI, NANO SEM430, Hillsboro, OR, USA). The microstructures of the composite PCMs were detected by transmission electron microscopy (TEM, JEOLJEM-2100, akishima, Tokyo, Japan) with an accelerating voltage of 200 kV. The phase change behavior of the samples was determined using a differential scanning calorimeter (DSC; Netzsch 200f3, Selbu, Bayern, Germany) in the range of 0 to 60 °C at a heating or cooling rate of 5 °C/min under nitrogen atmosphere.

The supercooling degrees of the samples (S-PCMs, GS-PCMs-1, GS-PCMs-2 and GS-PCMs-3) were tested using a multichannel temperature measuring instrument equipped with a computer, T-type thermocouple and thermostatic water bath. A 30 mL test tube filled with a 30 g sample was put into the thermostatic water bath and heated to about 45 °C; then, the sample was cooled from 45 to 10 °C. The temperature variations of the samples during the process were recorded to obtain the step cooling curves.

The thermal conductivity of the composite PCMs was measured by means of a hot disk thermal conductivity instrument (DRE-2C, Xiangtan Xiangyi Instrument Co., Ltd., Xiangtan, China) based on the transient plane source method and using a probe with a diameter of about 20 mm. Before the test, the probe was placed between two slabs of each sample and compacted. The thickness of each sample was not less than 10 mm, and each sample was tested three times to obtain the average value. The thermal conductivity *λ* was calculated as follows:

*λ* = *α*·*c*·*ρ*

where *α* represents the thermal diffusivity of a sample; *c* represents the specific heat capacity of a sample; and *ρ* indicates the density of a sample.

## 3. Results

### 3.1. Chemical Structure

**Figure 1:**
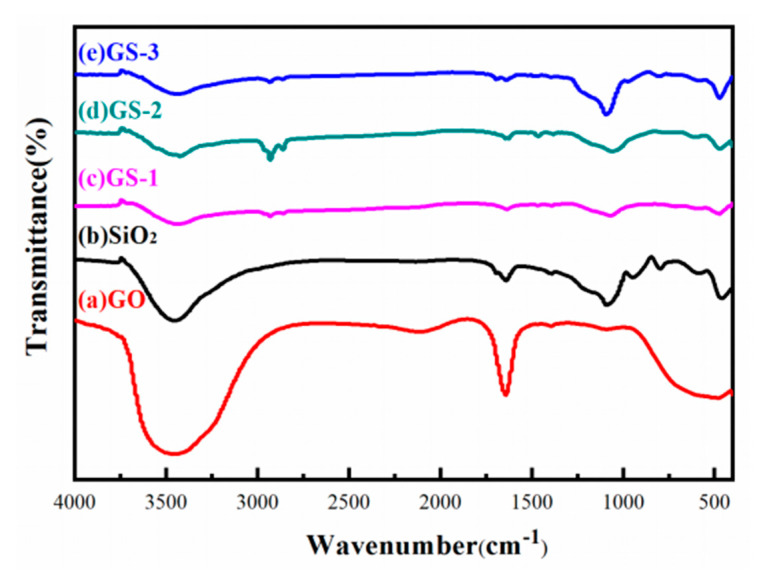
Fourier-transform infrared (FTIR) spectra of GO, SiO_2,_ GS-1, GS-2 and GS-3.

The FTIR spectra of SiO_2_, GO and GO–SiO_2_ are shown in Figure 1. In the spectrum for SiO_2_, the characteristic wide bands at 3000 cm^−1^ and 3700 cm^−1^ correspond to the stretching vibrations of –O–H, and the peak at 1640 cm^−1^ corresponds to the vibrations of H–O–H. The absorption peaks at 1051 cm^−1^ refer to the asymmetrical vibrations of Si–O–Si, while the peaks at 787 cm^−1^ and 451 cm^−1^ refer to the symmetrical vibrations of Si–O–Si. In the spectrum for GO, the bands at 3431 cm^−1^ are associated with the O–H stretching vibrations, and the peaks at 1640 cm^−1^ represent the bending vibrations of C–OH. The characteristic absorption peak at 1115 cm^−1^ corresponds to the C–O–C stretching vibrations. The results show that the oxygen-containing groups of GO are mainly hydroxyl(–OH), carboxyl(–COOH) and epoxy groups(C–O–C). The absorption peaks of SiO_2_ and GO can be clearly seen from the spectrum of the prepared GO–SiO_2_. No other new absorption peaks were present for the spectrum of GO–SiO_2_, which shows that there was no chemical reaction between the GO and SiO_2_ nanoparticles.

### 3.2. Micromorphology and Specific Surface Area Analysis

**Figure 2:**
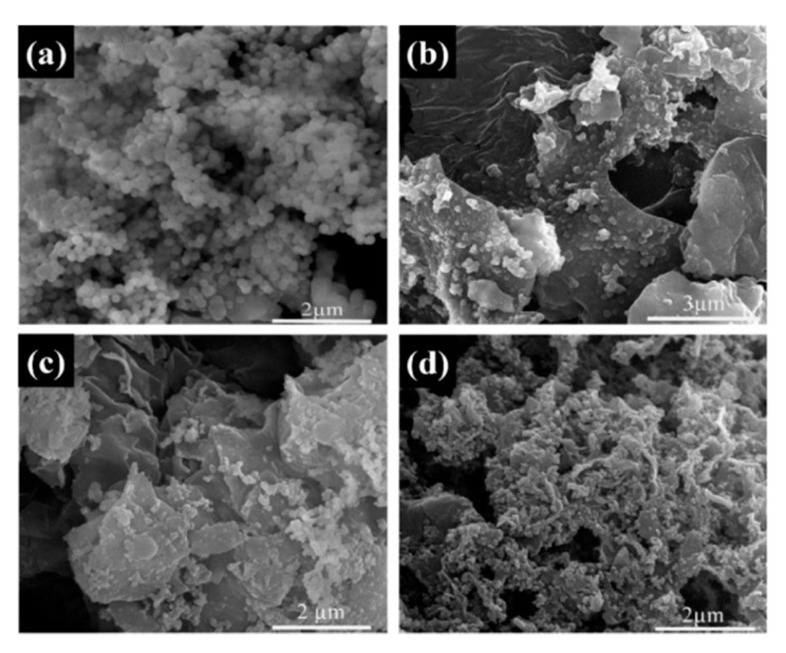
Scanning electron microscope (SEM) images of (**a**) SiO_2_, (**b**) GS-1, (**c**) GS-2, (**d**) GS-3.

The micromorphologies of the SiO_2_ and GO–SiO_2_ composites were observed by scanning electron microscope (SEM). As shown in Figure 2a, the spherical shape of SiO_2_ nanoparticles was easily to agglomerate together, with an average particle size of about 200 nm. In Figure 2b–d, showing the GO–SiO_2_ composites, the nanosheet structure of GO on which the SiO_2_ nanoparticles were absorbed can be clearly seen. As the amount of GO in the GO–SiO_2_ composites increased, the adsorption amount of the SiO_2_ nanoparticles obviously also increased (Figure 2d), indicating the good compatibility between the GO and the SiO_2_ nanoparticles [28,29]. Compared with the synthesized SiO_2_ nanoparticles, with the intercalation of GO, the agglomeration degree of the SiO_2_ nanoparticles was obviously reduced, while the hybrid network structure of GO–SiO_2_ increased the contact surface with the hydrate salts, which is helpful for the crystallization and nucleation of inorganic hydrate salts.

**Figure 3:**
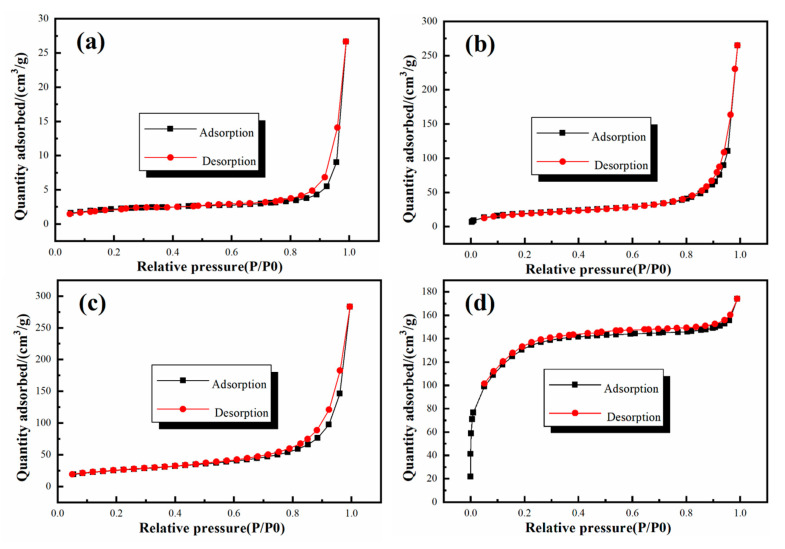
Nitrogen adsorption–desorption isotherms of (**a**) SiO_2_, (**b**) GS-1, (**c**) GS-2, (**d**) GS-3.

The nitrogen adsorption–desorption isotherms of SiO_2_ and GO–SiO_2_ are shown in Figure 3. The SiO_2_ nanoparticles presented a small specific surface area of about 27.53 m^2^/g. With the addition of GO, the specific surface areas of GS-1, GS-2 and GS-3 were increased to about 67.21 m^2^/g, 89.30 m^2^/g and 437.39 m^2^/g, respectively. A typical type Ⅳ isotherm with a hysteresis loop characteristic of relative pressure (*P/P*_0_) higher than 0.8 was observed, and it can be noted that the formation of the pore structure was caused by the accumulation of the nanoparticles. As the amount of GO increased, more and more pores were formed by the accumulation of the nanoparticles, meaning that the adsorption performance of GO–SiO_2_ was improved and the hydrate salts could be well adsorbed in the inner space of GO–SiO_2_.

### 3.3. Supercooling Degrees of the Na_2_SO_4_·10H_2_O Composites

**Figure 4:**
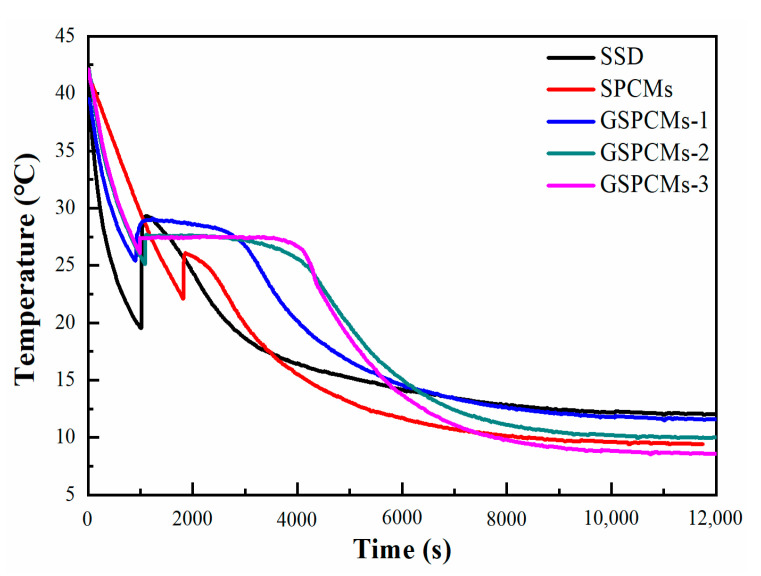
Step cooling curves of the pure Na_2_SO_4_·10H_2_O, S-PCMs and GS-PCMs.

The step cooling curves of the pure Na_2_SO_4_·10H_2_O, S-PCMs and GS-PCMs are shown in Figure 4. The supercooling degree of the Na_2_SO_4_·10H_2_O was reduced from 10.2 to 3.7 °C when SiO_2_ was added. SiO_2_ acted as the nucleating core, and the heterogeneous nucleation effect promoted the crystallization of the hydrate salts. For the GO–SiO_2_/Na_2_SO_4_·10H_2_O composites, the addition of GO further reduced the supercooling degree of the hydrate salts; with an increased amount of GO, supercooling degrees of about 3.3 °C, 1.9 °C and 1.2 °C were achieved for GS-PCMs-1, GS-PCMs-2 and GS-PCMs-3, respectively. The results show that the nucleation effect of GO–SiO_2_ was further enhanced with the introduction of GO. The increased surface area of GO–SiO_2_ provided a larger nucleation site for the hydrate salts, such that the crystallization of the Na_2_SO_4_·10H_2_O could be induced at a lower supercooling degree.

### 3.4. Crystallization Behavior

**Figure 5:**
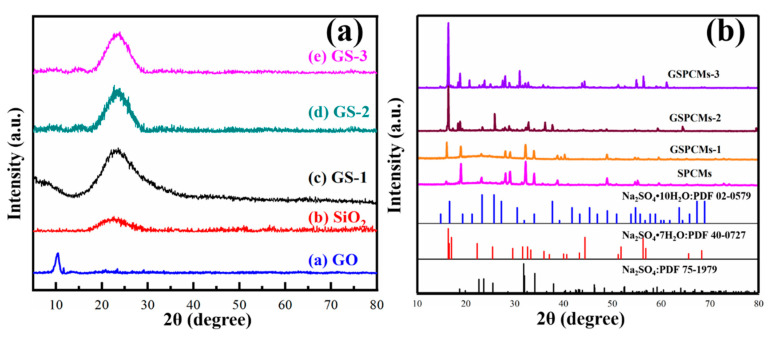
X-ray powder diffractometer (XRD) patterns of (**a**) GO, SiO_2,_ GS-1, GS-2 and GS-3; (**b**) Na_2_SO_4_·10H_2_O, S-PCMs, GS-PCMs-1, GS-PCMs-2 and GS-PCMs-3.

The XRD patterns of GO, GO–SiO_2_ and the GS-PCMs are shown in Figure 5. As can be seen from Figure 5a, a sharp diffraction peak of (002) was observed in GO around 2θ = 10.4°, which can be ascribed to the oxygen-containing functional groups contained in the nanosheet structure of GO. The broad diffraction peak at 2θ = 23.1° is a peak characteristic of SiO_2_, indicating the amorphous characteristics of SiO_2_. The diffraction peaks of GO and SiO_2_ can be seen for the GO–SiO_2_ composites. The crystallization peaks of pure Na_2_SO_4_·10H_2_O and of the composite PCMs are shown in Figure 5b. The diffraction peaks at 19.195°, 23.022° and 25.802° correspond to the crystallization peaks of Na_2_SO_4_·10H_2_O. For the S-PCMs that used SiO_2_ as a nucleating agent, diffraction peaks of Na_2_SO_4_ appearing at 23.022°, 39.133° and 60.457° can be observed, while weak diffraction peaks of Na_2_SO_4_·10H_2_O appearing at 19.195°, 23.022° and 25.802° can hardly be detected, indicating the dehydration of Na_2_SO_4_·10H_2_O in the phase change process. The diffraction peaks of Na_2_SO_4_·10H_2_O can be clearly seen from the XRD patterns of the GS-PCMs. It can thus be seen that the incorporation of GO improved the nucleation effect of SiO_2_, resulting in an enhanced crystallization performance for the Na_2_SO_4_·10H_2_O PCMs.

### 3.5. Phase Change Performance of the Composite PCMs

**Figure 6:**
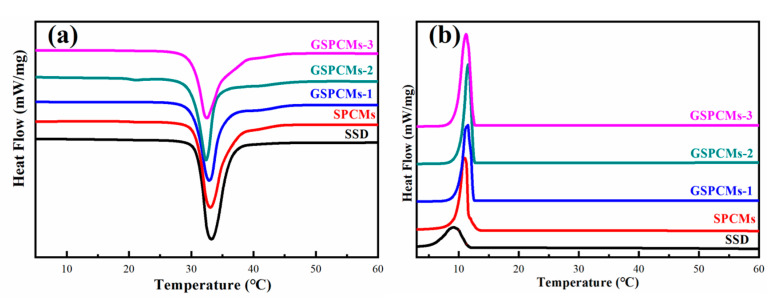
Differential scanning calorimeter (DSC) curves of Na_2_SO_4_·10H_2_O, S-PCMs and GS-PCMs. (**a**) melting process, (**b**) cooling process.

The phase change performances of the S-PCMs and GS-PCMs were determined using a DSC. The obtained curves in the melting and crystallization process are shown in Figure 6. The phase change performances of Na_2_SO_4_·10H_2_O and of the composite PCMs are shown in Table 2. The melting enthalpy of the pure Na_2_SO_4_·10H_2_O was about 208.2 J/g, with a phase change temperature of about 33.1 °C. The crystallization enthalpy of Na_2_SO_4_·10H_2_O was only about 67.4 J/g, indicating the poor crystallization performance of the pure Na_2_SO_4_·10H_2_O. The addition of SiO_2_ and GO–SiO_2_ had little influence on the phase change temperature of Na_2_SO_4_·10H_2_O. It can be noted that the crystallization enthalpy of the GS-PCMs obviously increased compared to the pure Na_2_SO_4_·10H_2_O. As the amount of GO in the GO–SiO_2_ composites increased, the phase change performance of the Na_2_SO_4_·10H_2_O composite PCMs greatly improved. Among the GO–SiO_2_ composite PCMs, GS-PCMs-3 possessed both the highest melting enthalpy, i.e., about 182.7 J/g, and the highest crystallization enthalpy, i.e., about 163.4 J/g.

### 3.6. Microstructure Analysis of Na_2_SO_4_·10H_2_O Composite PCMs

**Figure 7:**
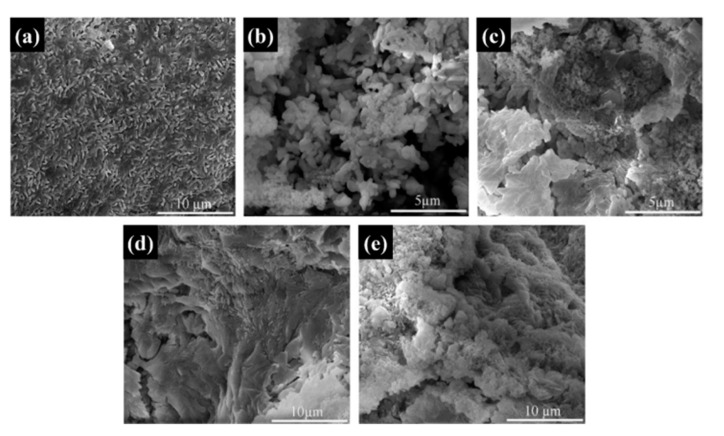
SEM images of (**a**) pure Na_2_SO_4_·10H_2_O, (**b**) S-PCMs, (**c**) GS-PCMs-1, (**d**) GS-PCMs-2, (**e**) GS-PCMs-3.

**Figure 8:**
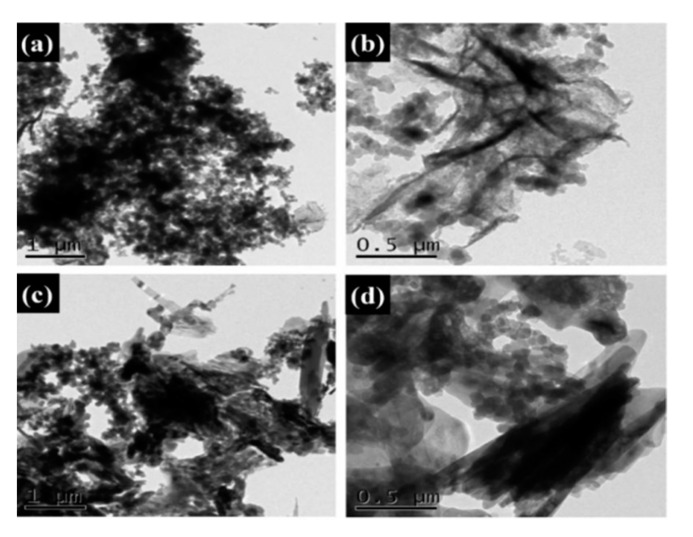
Transmission electron microscopy (TEM) images of (**a**,**b**) GO–SiO_2_(GS-3) and (**c**,**d**) GS-PCMs-3.

The microstructures of the Na_2_SO_4_·10H_2_O composite PCMs were tested using SEM. The fine crystalline grains of the pure Na_2_SO_4_·10H_2_O are shown in Figure 7a. As seen from Figure 7b, the addition of SiO_2_ resulted in coarse grain particles of Na_2_SO_4_·10H_2_O, indicating the weak nucleation effect of SiO_2_ that led to the irregular growth of the hydrate salts. Furthermore, to some extent, the change of the grain size also led to the reduction of phase change performance due to the existing phase transition of Na_2_SO_4_·10H_2_O to Na_2_SO_4_·7H_2_O or Na_2_SO_4_. In Figure 7c–e, the GS-PCMs with more compact structures can be seen; no coarse grain particles were found in these structures, indicating a better packaging efficiency for GO–SiO_2_ composites. It can also be seen that the introduction of GO–SiO_2_ refined the grain particle size and improved the crystallization behavior of the hydrate salts. The good compatibility of the hydrophilic GO implied a better combination of SiO_2_ with the hydrate salts, which further facilitated the nucleation and crystallization of the Na_2_SO_4_·10H_2_O PCMs [30,31]. The microstructures of the prepared GO–SiO_2_ (GS-3) and Na_2_SO_4_·10H_2_O composite PCMs (GS-PCMs-3) were observed by TEM. It can be clearly seen from Figure 8a,b that the spherical SiO_2_ nanoparticles were successfully absorbed on the surface of GO, indicating the successful formation of the GO–SiO_2_ composites. Figure 8c,d show the successful preparation of GO–SiO_2_/Na_2_SO_4_·10H_2_O composite PCMs; the GO–SiO_2_ composite in the Na_2_SO_4_·10H_2_O ensured a better nucleation effect, and the phase change performance of the composite PCMs was also improved.

### 3.7. Thermal Conductivity of the Composite PCMs

**Figure 9:**
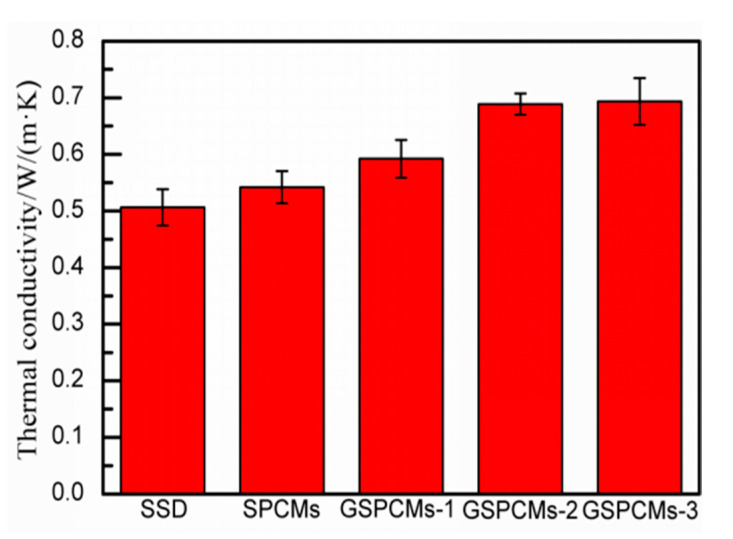
Thermal conductivity of the prepared S-PCMs and GS-PCMs.

The thermal conductivity of the prepared S-PCMs and GS-PCMs is shown in Figure 9. The test values of the pure Na_2_SO_4_·10H_2_O, S-PCMs, GS-PCMs-1, GS-PCMs-2 and GS-PCMs-3 were 0.5061 W/(m·K), 0.5419 W/(m·K), 0.5923 W/(m·K), 0.6887 W/(m·K) and 0.6934 W/(m·K), respectively. With the incorporation of GO in the GS-PCMs, the heat transfer performance of the GS-PCMs was enhanced as the specific area of GO–SiO_2_. Owing to the incorporation of GO–SiO_2_ in Na_2_SO_4_·10H_2_O, the latter was well dispersed and its heat transfer performance enhanced, with higher observed thermal conductivity of the GO–SiO_2_ materials. For the prepared sample, the GS-PCMs-3 preserved the highest thermal conductivity, i.e., more than 0.65 W/(m·K), indicating an improvement in the thermal conductivity of the GS-PCMs compared with pure Na_2_SO_4_·10H_2_O.

## 4. Conclusions

SiO_2_ nanoparticles was prepared and composited with GO to form GO–SiO_2_ composite materials with a layered structure. The GO–SiO_2_ composites were employed as nucleating agents in the Na_2_SO_4_·10H_2_O PCMs, and the influence of GO on the supercooling degree, crystallization behavior, phase change performance and thermal conductivity of the Na_2_SO_4_·10H_2_O composite PCMs was investigated. The following conclusions can be drawn:

(1) Compared to pure SiO_2,_ the dispersion performance of the SiO_2_ nanoparticles was obviously improved with the introduction of GO, and the specific area of GO–SiO_2_ was greatly increased. The high specific area of GO–SiO_2_ enabled a good nucleation effect and produced better crystallization behavior for the Na_2_SO_4_·10H_2_O PCMs. In comparison to pure Na_2_SO_4_·10H_2_O, the supercooling degree of Na_2_SO_4_·10H_2_O composite PCMs could be reduced from 10.2 to as low as 1.2 °C when 0.45 wt% of GO was added.
(2) The phase change performance of Na_2_SO_4_·10H_2_O was improved with the incorporation of GO–SiO_2_ composites. The crystallization enthalpy of the composite PCMs increased to about 163.4 J/g with the addition of 2.45 wt% of GO–SiO_2_.
(3) The good compatibility of the hydrophilic GO led to a better combination of SiO_2_ with the hydrate salts, which further facilitated the nucleation and crystallization of Na_2_SO_4_·10H_2_O. Compared with Na_2_SO_4_·10H_2_O, the thermal conductivity of the composite PCMs was improved due to the large specific area of the GO–SiO_2_ composites.
(4) The weight ratio of 2.45 wt% of GO–SiO_2_ in the Na_2_SO_4_·10H_2_O was appropriate for achieving a small supercooling degree, high latent heat of the phase change and high thermal conductivity. The prepared GO–SiO_2_ composited hydrated salt PCMs demonstrated potential applications in the domain of thermal energy storage materials.

## Figures and Tables

**Table 1:** Mix composition of the GO–SiO_2_ composites and the composite phase change materials (PCMs).

Samples	GO/ml	SiO_2_/g	Na_2_SO_4_·10H_2_O/g
GO–SiO_2_(GS-1)	20	2	-
GO–SiO_2_(GS-2)	40	2	-
GO–SiO_2_(GS-3)	60	2	-
S-PCMs	-	2	100
GS-PCMs-1	20	2	100
GS-PCMs-2	40	2	100
GS-PCMS-3	60	2	100

**Table 2:** Phase change performances of the pure Na_2_SO_4_·10H_2_O and composite PCMs.

Sample	Melting		Crystallization	
	Temperature (°C)	Melting Enthalpy (J/g)	Temperature (°C)	Crystallization Enthalpy (J/g)
Na_2_SO_4_·10H_2_O	33.1	208.2	9.4	67.4
S-PCMs	32.8	199.2	10.9	110.8
GS-PCMs-1	32.8	184.5	11.5	138.2
GS-PCMs-2	32.2	168.4	11.2	142.2
GS-PCMs-3	32.5	182.7	11.5	163.4
